# Returning to Work after Childbirth in Europe: Well-Being, Work-Life Balance, and the Interplay of Supervisor Support

**DOI:** 10.3389/fpsyg.2018.00068

**Published:** 2018-02-06

**Authors:** Ana M. Lucia-Casademunt, Antonia M. García-Cabrera, Laura Padilla-Angulo, Deybbi Cuéllar-Molina

**Affiliations:** ^1^Department of Business Administration and Department of Economics, Universidad Loyola Andalucía, Seville, Spain; ^2^Department of Business Administration, University of Las Palmas de Gran Canaria, Las Palmas de Gran Canaria, Spain

**Keywords:** childbirth, well-being in the workplace, female workers, European Union, work-life balance

## Abstract

Parents returning to work after the arrival of a new son or daughter is an important question for understanding the trajectory of people's lives and professional careers amid current debates about gender equality and work-life balance (WLB). Interestingly, current research concludes that general WLB practices at the workplace may be necessary in the specific case of women returning to work after childbirth because of the particular maternal and infant factors involved. However, WLB practices as a flexible arrangement may work against women because they may be viewed as a lack of organizational commitment. Therefore, research on this topic could benefit from considering supervisor support as a complement of such practices, but previous research has analyzed WLB and supervisor support separately and scarcely. To fill this gap in the literature, we use two sub-samples of 664 female employees and 749 male employees with children under the age of one from 27 European countries participating in the 6th European Working Conditions Survey (EWCS-2015) to study the impact of *perceived* WLB on European women's *perceived* well-being after childbirth, in contrast with previous literature. We also analyze the impact of perceived supervisor support (SS) and its interaction with perceived WLB on women's well-being after childbirth, and explore differences with men after childbirth, a collective underexplored by the literature. We find significant gender differences on the relative impact of WLB, SS, and their interaction on perceived job well-being. Our results have important implications for human resource practices in organizations. In particular, they suggest that gendered WLB practices should be encouraged, and stress the relevance of the human factor over human resource practices in addressing the difficulties that women returning to work face after childbirth.

## Introduction

Parents returning to work after childbirth is an important question for understanding the trajectory of people's lives and professional careers amid current debates about gender equality and work-life balance (WLB); being WLB understood as satisfaction and good functioning at work and at home with minimal conflict between parents' roles at both domains (Clark, 2000).

In the case of women, the benefits of they being employed are broad, including improvements in their mental and physical health, social support, and financial resources (Coulson et al., 2012), and many countries have become aware of the need to create conditions that facilitate women's abilities to combine their working and family lives (Kreyenfeld, 2010). At the organization level, WLB is also appreciated because work-family conflict will lead women to a re-evaluation of priorities, decreased organizational commitment (Fitzenberger et al., 2016), and harmed job-related well-being (henceforth, job well-being). Job well-being can be seen as an indicator of quality of working lives, e.g., occupational health (Schulte and Vainio, 2010), and a relevant determinant of performance at the individual or organizational levels, e.g., productivity and absenteeism (Boyd, 1997). Well-being in the workplace is defined as satisfaction with work and non-work domains, as well as general health (Aazami et al., 2015). Therefore, well-being is relevant for both employees and their organizations.

Nowadays, more than men, women quite often face demanding conditions to return to work after childbirth because of the specific maternal and infant factors involved (e.g., childcare, high women responsibilities with childcare, change of women's habits, daily physical and emotional effort to conciliate duties at home and work). Therefore, general conditions offered by governments aimed at reconciling working and family responsibilities may be insufficient and some firms may have an incentive to complement them applying suitable policies and practices, but this is not always usual. Therefore, there exists a relevant social concern about the issue, resulting in a rise in interest in maternal employment among academics (Skouteris et al., 2007), as well as journalists, editors, and other contributors covering highlights, articles, and tribune sections on this topic.

For example, Ms. Carme Chacón made it to the front pages and headlines of many international newspapers in 2008 when she became Spain's first female Defense Minister. Doubts emerged regarding her capacity to carry out her responsibilities as a minister while providing the attention a newborn requires and helping to raise her child. However, we can now say that Ms Chacón, who died from a congenital heart condition in 2017 at the early age of 46, may be remembered as a symbol of the reconciliation of family and work duties. Indeed, she accepted the charge to the Defense ministry in part because this governmental department had its own kindergarten^1^. Additionally, she was highly supported by the President of the government, and her defense duties were temporarily taken over by the Interior Minister after childbirth. However, although Spanish law allows for 16 weeks of maternity leave (paid time off), she was back at work within 6 weeks.

Obviously, the circumstances of this case would not have been news if the person affected by the official appointment and the forthcoming newborn was a man, showing once again that WLB is still a problem that profoundly affects women. This case may be unrepresentative of the majority of female employees, but illustrates the cost of motherhood in professional careers with high aspirations, as well as the relevance of superior support and the existence of conditions to facilitate WLB (e.g., the kindergarten) to help women return to work. In this case, they were the crucial factors that allowed Ms. Chacón to bring her child up and retain her post as Minister. In developing this professional role, although she felt some “sensation of guilt that moms have when their work is intense,” she was sufficiently vigorous and in good spirits to work in order to fulfill her dreams and job goals^2^. In other words, she demonstrated job well-being, which, according to Boyd (1997), is quite relevant to making good contributions at work. Although this case illustrates that reconciliation is possible, conditions that allow women to properly balance work and family life duties after childbirth and to enhance their actual performance and perception of well-being at work are not always present in firms. The current study aims to contribute to this area of research.

Although motherhood represents one of the most important stages in women's lives and is quite relevant in societies, women still encounter diverse difficulties after becoming mothers, even during the first 3 years postpartum (Hsu and Wickrama, 2017). While motherhood is often considered a positive and fulfilling experience (Umberson et al., 2010), in some cases, it is more difficult than expected and eventually perceived as a negative experience (Nichols and Roux, 2004) because it involves facing new situations that are potentially stressful, e.g., childcare, change of habits, and personal time (Fisher and Stocky, 2003). All of a sudden, working mothers have to simultaneously manage a job, raise children, and maintain a home involving a challenging physical and emotional effort (Maynard and Blain, 2005). Moreover, mothers commonly experience fatigue, sleep-related problems, and in some cases, high levels of stress and depression (Maynard and Blain, 2005). Consequently, women can become highly vulnerable in face of the potential conflict between the roles of mother and employee.

Hence, the return to work after childbirth does not always involve an adequate performance level of new mothers, particularly when there is lack of engagement from the employers regarding WLB (Guendelman et al., 2014). Therefore, managing the return to work of women after childbirth is a challenge for the human resources departments of companies (Kossek et al., 2010). For example, flexible work arrangements are considered suitable practices (Lee et al., 2002).

Previous research has investigated women's experiences balancing work and family life after childbirth from different perspectives (Grice et al., 2008, 2011, 2017). For example, some studies have shown that work conditions related to WLB that are established by firms can affect maternal postpartum mental health (Cooklin et al., 2011; Coulson et al., 2012) and well-being (Cooklin et al., 2011). However, managers' opinions on the practices being implemented can be quite different from the employees' views about such practices (e.g., Khilji and Wang, 2006), and as a consequence Farndale and Paauwe (2007) recommended the study of conditions implemented by firms from the perception of employees. However, the link between women's perceptions of WLB practices offered by their firms and job well-being following childbirth has been under-examined. To better understand how women address their return to work, we try to fill this gap in the literature by analyzing the impact of women's perceived WLB in their firms on job well-being after childbirth.

In addition, since women's use of WLB alternatives such as flexible arrangement formally offered by the firm might also work against them because being considered as showing lack of organizational commitment (Woolnough and Redshaw, 2016), research on this topic could benefit from considering postulates of the theory of perceived organizational support (Eisenberger et al., 1986). This theory posits that employees develop beliefs regarding the extent to which the company cares about their well-being and values their actual contributions (e.g., not being evaluated solely based on their use of a flexible arrangement). The SS is clearly a good indicator of company support (Boselie et al., 2005). We took into account this theory and studied the SS, as well as its potential moderating role in the effect of WLB on women's job well-beings. Certainly, it can be expected that supervisors' opinions, whether positive or negative, about women's use of existing WLB alternatives may be relevant for women, as these opinions may condition the colleagues' views and the social support women experience after returning to work.

The present study addresses the scarcity of research on the impact of employed postpartum women's perceptions of WLB on their job well-being. In particular, previous and scarce research has analyzed the impact of WLB and SS separately on women's general mental health after childbirth (Grice et al., 2008) and the impact of perceived work-schedule flexibility on the general well-being of working parents with children under age 18 (Jang, 2009). In this research, well-being was essentially measured by stress-related symptoms. Therefore, to the best of our knowledge, we are the first to analyze the impact of the perception of WLB, SS, and their interaction, on women's perceptions of job well-being in the particular setting of having with children under the age of one's perceptions of job well-being. Moreover, we extended this analysis to men after childbirth. On this basis, the current study aimed to analyze the following: (a) the influence of the perceived WLB and SS on women work-related outcomes such as job well-being after childbirth, and (b) the moderating effect of SS on the effect of WLB on women's job well-beings, in both cases comparing women with men to find qualified results. We analyzed two sub-samples of 664 working women and 749 working men with children under the age of one in 27 European countries. The use of these two sub-samples was highly appropriate because it allowed the study of the specific conditions that affect the relationships between the studied variables for women in comparison to men, thus assuring that results did not only reflect what any employee needs at work but also illustrate the specific requirements of the collective under study.

## Theoretical background

### Women's job well-beings after childbirth

Over the past few years, employee well-being has occupied an increasingly prominent place in organizational psychology. Research on well-being is a broad domain that has flourished in recent decades (Keyes et al., 2002); however, there is no academic consensus on its definition and measurement (Franco-Santos and Doherty, 2017). In the current research, job-related well-being is defined as the global quality of an employee's experience and functioning at work (Guest, 2017) distinguishes three facets of job-related well-being, which are related to psychological, physical, and social functioning. We adopt the psychological approach, extensively and successfully used across a wide range of study fields (Topp et al., 2015). The psychological approach distinguishes between “hedonic” and the “eudemonic” well-being (Robertson and Cooper, 2011). On one hand, the term “hedonic” well-being is defined in terms of pleasure attainment and pain avoidance and is used to refer to subjective feelings of happiness. On the other hand, the eudemonic approach focuses on meaning and self-realization and is generally used to refer to the degree to which a person is fully functioning (Schulte and Vainio, 2010). We adopt the eudemonic perspective of well-being, that is, psychological well-being (PWB). According to Warr (1987), well-being is composed of three components—enthusiasm, pleasure or serenity, and finally, vitality or strength. These three components are based on Warr's (1987) model and are used by the World Health Organization (WHO), regional office for Europe (1998) to elaborate a well-being Index based on the eudemonic approach.

Managers can influence their employees' job well-beings by changing dimensions of organizational contexts, such as working hours, tasks, or rewards (Danna and Griffin, 1999). Both employees and employers may benefit from increased job well-being. Specifically, employees that experience poor well-being are also less productive, make worse quality decisions, and are more prone to absenteeism (Boyd, 1997); hence, the company suffers from decreased performance.

In the case of female employees, evidence suggests that there is a need to provide women with support in order to increase their job well-being, especially at times that are highly demanding (e.g., childbirth, caring for an aging parent). In this respect, the SS or the increased provision of alternative work arrangements that facilitate the combination of their working and family life (i.e., work-life balance can be relevant). Indeed, both SS and WLB policies and practices are indistinctly understood as workplace interventions, representing organizational efforts to improve job well-being through the provision of services and resources (e.g., employee assistance programs). Although these services are examined extensively elsewhere, they have been noticeably ignored in this area of study (Grandey et al., 2007). Therefore, in order to test previous premises, our research focuses on the impact of WLB and SS on a highly relevant work-related outcome strongly associated with mothers' postpartum health, such as women's perceived job well-beings after childbirth.

### Work-life balance and women's perceived job well-beings after childbirth

WLB can be defined as “satisfaction and good functioning at work and at home with a minimum amount of role conflict” (Clark, 2000, p. 349) or the ability to allow “sufficient time to meet commitments at both work and home” (Guest and Conway, 2002, p. 263).

The relationship between WLB and well-being is well documented in the literature. Previous research finds that balance between work and family predicts well-being and overall quality of life (Greenhaus et al., 2003) and helps employees feel better (Gröpel and Kuhl, 2009). Conversely, work-family conflict is associated with reduced job and life satisfaction (Hsu, 2011) and decreased well-being and quality of life (Gröpel and Kuhl, 2009).

Although the balance of work-family demands affects both parents, women generally shoulder the majority of family responsibilities, particularly after childbirth (Grice et al., 2011). As already mentioned, previous studies have investigated women's experiences balancing work and family after childbirth from different perspectives (Killien et al., 2001; Grice et al., 2011, 2017), but the specific link between perceived WLB and job well-being has been under-examined for women after the birth of a child.

An often-used framework for describing how multiple roles are balanced is the role stress theory (Van Hooff et al., 2005). This theory posits that people work under time, energy, and psychological constraints and that they draw energy from finite resources. Adding roles may lead to increased burden since resources are fixed. Surpassing time, energy, or psychological limits may result in overload and, ultimately, conflict (Grant-Vallone and Donaldson, 2001). As mentioned above, after childbirth, employed mothers generally commit relatively more time to family responsibilities than employed fathers, and this increase in role strain may result in poorer well-being (Grice et al., 2011). Therefore, based on the role stress theory, we propose the following hypothesis:

*H1: The greater the perceived WLB is, the greater the job well-being of women after childbirth*.

### Supervisory support and women's perceived job well-beings after childbirth

Previous research has shown that SS also helps to increase employee's job satisfaction, well-being, and health (Marcinkus et al., 2007; Di Fabio et al., 2017).

The theory of perceived organizational support (POS) (Eisenberger et al., 1986) helps to explain this link between the behavior of managers and employees' job attitudes and well-beings (Rhoades and Eisenberger, 2002). The POS theory says that employees develop beliefs regarding the extent to which the company cares about their well-being and values their contributions, and SS is considered by employees to be an indicator of POS (Boselie et al., 2005). For most employees, the personification of the firm is the immediate supervisor (Levinson, 1965) because employees have direct contact with the supervisor. It is expected, then, that the perception of employees about the support they receive from their supervisor will affect their job well-being.

Indeed, previous research has shown that employees who perceive their firm as supportive of family experience more job well-being (Marcinkus et al., 2007). Since SS is one of the most direct means that companies have to demonstrate a family-supportive culture, studies have equated support from the immediate supervisor with family-supportive practices and have considered such support a part of “family friendliness” (Marcinkus et al., 2007). Accordingly, we state that women returning to work after childbirth will be particularly sensitive to SS given the unique challenges they face, as explained above. Thus, basing on the POS theory, we propose the following hypothesis:

*H2: The greater the perception of SS is, the greater the perceived job well-being of women after childbirth*.

### Supervisory support as a moderator in the influence of perceived WLB on women's job well-beings after childbirth

Previous studies have shown that women who access WLB alternatives formally offered by the firm experience prejudice because they are seen as lacking in commitment to the firm (Brown, 2010). As a consequence, the professional careers of women tend to stagnate after motherhood (Woolnough and Redshaw, 2016) and either the mere risk of this stagnation or women's willingness to simultaneously fulfill the usual conditions at work along with the increasing demands at home result in poorer job well-being. Hence, we can expect that not all female employees will react to the WLB alternatives offered by their firm in the same way, as other circumstances play a role in this process. In other words, not all women will experience increased well-being because of the mere existence of WLB practices in their firms. In this respect, the work-related social support may differ from one firm to another (e.g., awareness of women's challenges after childbirth, recognizing the possibility of women accessing WLB practices while still showing a good performance at work). This work-related social support might interfere with (i.e., moderate) the effect of the availability of WLB alternatives in the firm on women's job well-beings after childbirth.

To study this moderating role of work-related social support, we base our research on POS theory (Eisenberger et al., 1986) because it recognizes that firms and supervisors (Eisenberger et al., 1986) may provide different levels of support to employees, which is noticed by employees (Boselie et al., 2005). This argument can be seen as an extension of previous research that finds that work-based social support moderates the impact of work-family balance on job performance and satisfaction. For example, Hsu (2011) shows that, while work-family conflict (i.e., the lack of work-life balance) has a negative impact on job satisfaction in stressful working environments, perceived SS significantly moderates the relationship between work-family conflict and job satisfaction. Similarly, Wang et al. (2014) find that organizational support weakens the relationship between work-family conflict and the job performance of nurses. Furthermore, several studies allude to relationships between satisfaction with WLB and the role of support (Clark, 2000; Van Daalen et al., 2006). In many cases, these results can be explained through the impact of work-based social support on employees' stress levels. In effect, one of the most important negative consequences of work-family conflict is the generation of stress (Allen et al., 2000), and previous research has found that work-based social support moderates the effect that stressful situations have on employees' well-beings. For example, Baeriswyl et al. (2016) provided evidence of the positive impact of supervisor support on airport security officers in the case of increased workload after the recent, growing threat of terrorism, which affected their work-life balance.

In the particular case of women after childbirth, when examining SS, we can certainly expect that supervisors' opinions, whether positive or negative, about women's use of WLB options may be relevant for women considering accessing them, as well as for the supervisor transferring their opinions to colleagues. This idea is in line with findings from studies by Babin and Boles (1996) or Parasuraman et al. (1992), which found that supervisory support can help decrease work stress to provide employees with more job well-being.

In spite of previous arguments, there is a paucity of research investigating the interaction effect of SS and WLB, which may act as a buffer against the impact of women's burdens when returning to work after childbirth. We believe that women's feelings that their supervisor helps and supports them when they return to work after childbirth could help them take advantage of and maximize the positive impact that WLB practices can have on their job well-being. Consequently, we propose the following hypothesis:

*H3: The greater the SS is, the stronger the impact of perceived WLB on the job well-being of women after childbirth*.

## Research methodology

### Data sources and study context

Data were obtained from the 6th European Working Conditions Survey (EWCS) (Eurofond, 2012), carried out in 2015 by the European Foundation for the Improvement of Living and Working Conditions. Among other aspects, this survey addressed issues in the general job context, working conditions, work-related health risks, cognitive and psychosocial factors, work-life balance, and access to training. The target population in this study included workers aged 15 years and older (16 and older in Spain, the UK and Norway) who were employed in the country being surveyed. The total number of interviews in the EWCS (2010) was 43,816 in 27 EU Member States (except Switzerland, Ukraine, Czech Republic, and Russia), as well as some countries that were not yet members of the Union (Turkey, Croatia, Norway, Macedonia, Montenegro, Albania, and Kosovo). In light of the objective of this research, we obtained two sub-samples of 664 working women and 749 working men with children under the age of one, that is, mothers and fathers that after childbirth are conciliating work and childcare. The sizeable reduction in the sample was the result of selecting only employees in Europe with children under the age of one, as well as disregarding the self-employed.

From a demographic perspective, the sub-sample of men consisted of employees who were 33.43 years of age or younger (58.7%). With regard to their educational level, more than half of the respondents (30.2%) had reached the “upper secondary education” level, 60.9% in the private sector and 29.9% in the public sector. The largest percentage of employees (33.6%) was concentrated in medium-sized firms with 10–49 employees. With respect to the sub-sample of female managers, in which more than half of the individuals had reached the “upper secondary education” level (41.4%), they were, on average, 35.02 years old, with 74.4% belonging to the private sector and 19.9% belonging to the public sector. The largest percentage of female employees (32%) was concentrated in medium-sized firms with 10–49 employees.

### Measures

#### Dependent variable

We measure perceived job well-being by using a 5-item World Health Organization Well-Being Index (WHO-5) from the 6th EWCS (Eurofond, 2012). Specifically, we use items labeled 87a-e from the questionnaire. EWCS collected information on self-reported well-being and individuals' perceptions upon the state of mind, according to World Health Organization (WHO), regional office for Europe (1998) with the WHO-5 Well-Being Index based on the eudemonic approach. The WHO-5 well-being index has been validated in previous research (Wanous and Hudy, 2001). Interviewees were asked to indicate if over the previous 2 weeks they: (1) felt cheerful and in good spirits, (2) felt calm and relaxed, (3) felt active and vigorous, (4) woke up feeling fresh and rested, and (5) their daily life was filled with things that interest them (Robins et al., 2001). All these items were answered using a Likert-Type response scale with six response options, ranging from 1 to 6: at no time, some of the time, less than a half of the time, more than a half of the time, most of the time, and all of the time. The results showed that both the Kaiser-Meyer-Olkin (KMO) test and Bartlett's test of sphericity (χ^2^) offered satisfactory levels (KMO = 0.720, χ^2^ = 1508.515^***^). The variance explained rose to 73.76%. The Cronbach's alpha coefficients indicated that the scales used to measure employee well-being had internal consistency (0.819).

#### Independent variables

Two items were chosen from the 6th EWCS as proxies of the variables under study, as perceived by employees: SS and WLB. Although numerous researchers are in favor of the use of multiple-item measures, this trend has been challenged (Loo, 2002). In fact, there are numerous authors who have analyzed the validity of single-item measures, and their findings provide qualified support for them (Robins et al., 2001). Specifically, the use of a single-item scale for capturing the constructs under study has demonstrated the ability to validly predict outcomes (Wanous and Hudy, 2001). In the particular case of HRP through employees' perceptions as independent variables, our review of the empirical literature ratified the use of a single variable. Accordingly, SS was measured through the following question: “For the following statement, please select the response which best describes your work situation: Your manager helps and supports you” [Likert-type response scale ranging from “never” (1) to “always” (5)]. WLB was measured through the following question: “In general, how do your working hours fit in with your family or social commitments outside work?” [Likert-type response scale ranging from “not at all well” (1) to “very well” (4)].

#### Control variable

Two variables were treated as control variables in the multivariate analyzes to be reported: number of children and general health. We controlled for the number of children because previous research has indicated that this factor may confound the relationship between the study variables (Greenhaus et al., 2003). For example, more children at home have been associated with increased work and family demands, leading to higher levels of work-family conflict and consequently to reduced well-being (Perrewé et al., 1999). On the other hand, women experience poor mental and physical health during the postpartum period (Maynard and Blain, 2005), which affects well-being; consequently, it was significant to incorporate general health as control variable in our study. Respondents were asked: “How is your health in general? Would you say it is…,” and they rated their health on a scale ranging from “very bad” (1) to “very good” (5).

### Data analysis

First, bivariate correlations were examined among the variables job well-being, number of children, general health, WLB, and SS for the two sub-samples of working women and working men. These analyzes allowed us to examine the possibility of multicollinearity. Second, we estimated separated hierarchical regressions for the sub-samples of women and men. Specifically, multiple linear regression analyzes were conducted to study the direct effect of independent variables on job well-being, as well as the moderating effect of SS on the impact that WLB exerts on job well-being. The variables were entered in the following three steps: (1) controls (number of children and general health), (2) WLB and SS, and (3) the final step included one cross-product term representing interaction between work-life balance and supervisor support. To minimize the possibility of multicollinearity, we centered all main effect variables to calculate interaction term (Edwards and Lambert, 2007). The *F* statistic and adjusted *R*^2^ were calculated in each step, as β-values for all variables were introduced. In addition, collinearity diagnostics, i.e., variance inflation factor (VIF) scores and condition number, were conducted in order to assess the potential for regression coefficient instability. Finally, as this research is cross-sectional in nature and uses a single data source, it could result in common method variance. We ran Harman's one-factor test to assess the possibility of common method variance. Tests show that 2 factors had eigenvalues greater than 1 in the sub-sample of both women and men, regardless of whether the unrotated principal-component factor analysis (total variance explained = 54.41 and = 48.32%, respectively), maximum likelihood with varimax rotation (total variance explained = 37.53 and = 29.36%, respectively), or principal axis analysis with varimax rotation (total variance explained = 37.32 and = 29.25%, respectively) was used. For the female sub-sample, the first factor explained 39.45, 31.30, and 30.42% of the total variance. For the male sub-sample, the first factor explained 32.72, 23.24, and 23.10%. Hence, we did not assume common method variance to be a major concern in this study. These analyzes were all performed using SPSS version 21.0 (IBM Corp 2012).

## Results

Tables 1, 2 present the means, standard deviations, and correlations for the study variables for both sub-samples of employed mothers and employed fathers. General health, WLB, and SS were positively associated with job well-being for both employed women and men, whereas the interaction variable (work-life-balance × SS) had a negative and weak correlation with well-being for both sub-samples. Concerning multicollinearity in the data, the general rule of thumb is that correlation should not exceed 0.75 between independent variables (Tsui et al., 1995). Therefore, excluding correlations involving job well-being, the highest correlations were between general health and WLB, at 0.142 in the female sub-sample and 0.214 in the male sub-sample. These correlations suggest that multicollinearity was not a problem. In addition, our tests for hierarchical regressions (Table 3) showed that the variance inflation factor (VIF) values ranged from 1.006 to 1.076 in the female sub-sample and from 1.011 to 1.093 in the male sub-sample, both of which were much lower than the recommended cut-off threshold of 10 (Hair et al., 1992). The highest condition number for all the regressions in the female sub-sample was 18.174 lower than the recommended cut-off of 20 (Belsley, 1991) and reached 21.288 in the male sub-sample, slightly higher than this limit. These statistics suggest that multicollinearity was not a problem in the data.

**Table 1:** Correlations, means, and standard deviations (Employed Mother).

	**1**	**2**	**3**	**4**	**5**	**6**
1. Job well-being	1					
2. Number of children	−0.017	1				
3. General health	0.257^***^	−0.050	1			
4. Work-life balance	0.118^**^	−0.028	0.142^***^	1		
5. Supervisor support	0.200^***^	−0.043	0.105^*^	0.114^**^	1	
6. Work-life balance × Supervisor Support	−0.128^*^	0.046	−0.088	0.254^***^	0.092	1
Mean	−0.024	1.79	4.20	3.00	3.81	0.56
Standard deviation	0.95	0.93	0.69	0.75	1.16	0.87

*** *p < 0.001*,

** *p < 0.01*,

* *p < 0.05*.

**Table 2:** Correlations, means, and standard deviations (Employed Father).

	**1**	**2**	**3**	**4**	**5**	**6**
1. Job well-being	1					
2. Number of children	−0.013	1				
3. General health	0.321^***^	−0.098^*^	1			
4. Work-life balance	0.309^***^	−0.008	0.214^***^	1		
5. Supervisor support	0.237^***^	−0.008	0.107^*^	0.189^***^	1	
6. Work-life balance × Supervisor support	−0.067^*^	0.0132^*^	−0.126^*^	0.157^*^	0.004	1
Mean	0.020	1.78	4.16	2.86	3.78	0.38
Standard deviation	1.03	0.94	0.72	0.79	1.12	0.82

*** *p < 0.001*,

* *p < 0.05*.

**Table 3:** Estimated models and hypothesis tests (hierarchical regression).

**Variables**	**Model 1 Employed Mothers**	**Model 2 Employed Fathers**
	**β standardized**	**β standardized**
**STEP 1: CONTROLS**		
Number of children	0.135^*^	−0.071
General health	0.359^***^	0.174^**^
**STEP 2: CONTROLS + MAIN EFFECTS**		
Number of children	0.137^*^	−0.070
General health	0.314^***^	0.154^**^
Work-life balance	0.133^*^	−0.014
Supervisor support	0.169^**^	0.214^***^
Δ*R*^2^	5.4%	4.5%
ΔF	7.371^**^	6.607^**^
**STEP 3: CONTROLS + MAIN EFFECTS + INTERACTION EFFECT**		
Number of children	0.158^**^	−0.066
General health	0.295^***^	0.148^*^
Work-life balance	0.155^**^	0.004
Supervisor support	0.168^**^	0.219^***^
Work-life balance × Supervisor support	−0.132^*^	−0.071
Δ*R*^2^	1.6%	0.5%
ΔF	4.526^*^	1.380
*F*	11.637^***^	5.157^***^
Final adjusted *R*^2^	19.1%	7.1%
Durbin-Watson	1.912	1.919
Condition number	18.971	21.288
VIF	1.006–1.076	1.011–1.093

*** *p < 0.001*,

** *p < 0.01*,

* *p < 0.05*.

Table 3 shows the regressions estimated to analyze the direct and moderating effects proposed in the hypotheses. The two control variables had different effects in the equations estimated. First, since women may be vulnerable in terms of general health given their postpartum situation, their health was important for their job well-being (β = 0.359^***^). Even by 6 months postpartum, some women did not feel completely recovered from childbirth. Although general health was also relevant for men and positively conditioned their job well-being (β = 0.174^**^), for women, this relationship was doubly important. Moreover, the number of children, in addition to the newborn, was also relevant to the job well-being of women, and the higher the number of children is, the greater the perceived job well-being (β = 0.135^*^), but this variable did not affect men's job well-beings. This difference probably arose because experienced mothers have learned to organize the multiple tasks they must undertake in order to perform well at work and at home. This finding illustrates another reason to demand more resources that help to address the difficult postpartum situation.

Furthermore, our results offer additional evidence that employed women after childbirth had a higher need for organizational support through WLB practices and SS in order to increase their job well-being. Regarding the direct effects, the results confirmed hypotheses H1 and H2 and verified the relevance of WLB and SS on women's well-beings. Specifically, we identified significant positive expected effects (β = 0.155^**^ and 0.168^**^). Nevertheless, the effect of WLB on job well-being was non-significant in the male sub-sample probably because women are more involved in and committed to home life than men, and women need their firms to provide them with working hours that fit with their family requirements. This result suggests that it might be interesting to develop WLB practices based on gender.

**Figure 1:**
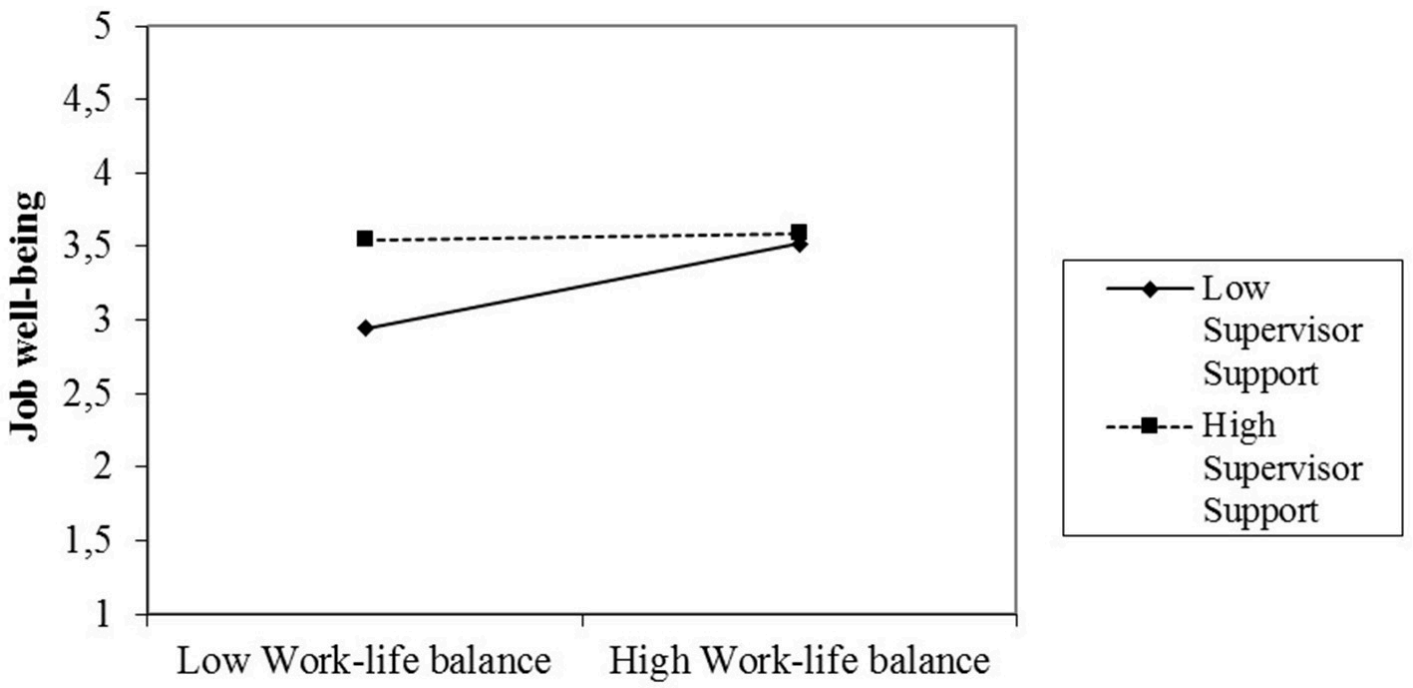
Plot of the two-way interaction effect of supervisor support and work-life balance on women's job well-being after childbirth.

Moreover, we found a significant and negative impact for the interaction effect of WLB and SS on job well-being (step 3 in Table 3), but this interaction effect exists only in the case of women (Figure 1) and was non-significant for the male sub-sample. This effect is the opposite of what we expected; thus, H3 was not supported. The negative interaction indicates that the joint effect of WLB practices and supervisor support is different before and after the cutoff point depicted in Figure 1.

The negative effect indicates that, before the cutoff point, supervisor support is highly relevant to increasing women's well-beings, although the firm provides opportunities to balance work and life. However, after the cut-off point, that is, in the presence of highly relevant WLB practices, the role of supervisor support is less important to increasing well-being.

## Discussion and conclusion

This study addressed two research objectives.

The first objective was to measure the direct impact of WLB and SS on the well-being of mothers and fathers with children under the age of one. Additionally, we studied the potential moderating role of SS on the effect that WLB exerts on job well-being for mothers and fathers. Consistent with previous studies on employees' well-beings, we found that WLB and SS positively influenced the well-beings of female employees after childbirth (e.g., Grice et al., 2008; Jang, 2009; Cooklin et al., 2011), but the effect of WLB on job well-being was non-significant for the men. These results offer evidence of the higher need among employees who are mothers for WLB practices in order to increase their job well-being when returning to work after childbirth. Accordingly, a practical implication of this study refers to the location of WLB and flexibility debates. Our results suggest that many times, such debate occurs within a gender-neutral context and should instead provide encouragement for gendered WLB practices, which are particularly suitable for a specific life stage such as returning to work after motherhood. Regarding SS, as both women and men need such work-related support, after the arrival of a new son or daughter, in order to facilitate returning to work, our study showed the relevant role that the supervisor has, rather than the firm's formalized organizational practices. Certainly, this finding stresses the relevance of the human factor over human resource practices in addressing the difficulties that parents returning to work face when they have children under the age of one. This conclusion emerges if SS and WLB practices are analyzed separately.

Regarding the SS moderating effect, SS was not found to affect the relationship between WLB practices and job well-being for men, but it moderated this relationship for women. The identified interaction was significant and negative, which is the opposite of what we expected on the base of literature that focus on the moderation effect of supervisor support and WLB on employees outcomes (Allen et al., 2000; Wang et al., 2014; Baeriswyl et al., 2016). Obviously, when a significant interaction is detected, its interpretation becomes critically important. In our case, the estimated interaction indicated that to enhance women's job well-beings after childbirth, the effect of SS differed depending on whether WLB practices were low or high. It is noteworthy that the joint effect of WLB practices and supervisor support is different before and after the cutoff point in Figure 1, which is in concordance with the negative terms of the interaction that we have found (Table 3).

Before the cut-off point, when women perceived low WLB practices, SS took on a greater role in increasing women's job well-beings. Therefore, the low use of WLB practices was somehow *compensated* by a supervisor who was supportive and receptive to female employees' family needs, which had a stronger effect on final well-being. In contrast, in cases of a high presence of WLB practices, the relevance of SS, although reinforcing and helping women access to these practices, were less relevant for the improvement of women's well-beings. Focusing on the case of low supervisor support, employee well-being benefits from higher levels of WLB practices. However, if a high level of supervisor support exists, increasing WLB practices has little effect on improving employee well-being.

After the cut-off point, higher levels of well-being can be reached in cases where the organization provides significant WLB practices, even with low existing supervisor support. In other words, supervisor support drops in relevance if the HRP is significant. Additionally, job well-being should reach higher values for women in cases of high WLB practices and some SS, suggesting that women felt more supported and confident in balancing job and family needs when that support is guaranteed by the firm's formalized WLB practices. It happens only after the cut-off point, because before it, the supervisor support is what mainly increases women's well-beings.

Some arguments may justify these results. First, if supervisor support is low, any increase in the number of WLB practices will improve well-being. However, even if supervisor support is high, the organization must place high importance on the application of these practices to improve women's well-beings because the supervisor can offer support to the woman only up to a point, from which greater flexibility should be allowed and even offered by the organization through formalized practices. From another point of view, when there is a certain lack of WLB practices, the supervisor becomes the main support for employed mothers with children under the age of one, as supervisors are close to these employees, interact with them and know first-hand the problems they face daily (e.g., days when children fall ill or do not sleep well). As such, these supervisors become the more relevant organizational medium to help employed mothers balance work and family life. The supervisor is then the only person that can actually offer alternatives to reach such balance and provide the help that really fits women's needs after childbirth. However, this happens till a given point, from which the supervisor has unlikely the authority to offer more support and flexibility to women.

On the basis of the identified SS moderating effect, the present study suggests that despite the increasingly normalized career orientation of females and the higher commitments of firms to ensure WLB, female employees require targeted and adapted WLB policies to return to work after childbirth, and this is likely to be possible only if SS exists, as the supervisor plays a relevant role in facilitating the returning to work in cases where the firm has not a high commitment to the offering of WLB practices. In addition, as the mere offer of some WLB alternatives by the firm may be insufficient for women who access such options because they face prejudices for being considered lacking in commitment to the firm and, consequently, harming their professional career, the support of their immediate supervisor becomes very relevant. The relevance of SS only declines in cases where the firm is firmly committed to the WLB policies.

New practical implications of this study emerged from the SS moderating effect. First, human resource managers and practitioners in firms in which women of childbearing age work should send clear signs to employees through different but simultaneously congruent human resource policies and practices that help them to face the uncertainty associated with their return to work after childbirth. For example, if flexible work arrangements are offered, female employees should not be viewed negatively if they benefit from them, and they should be clearly informed about existing alternatives and supported by supervisors to access to options to facilitate reconciliation and successfully perform their functions at work. Second, supervisors should be advised about their relevant role in the experience of returning to work for both female and male employees. Indeed, this role of supervisors is the only mechanism that our study determined to be relevant for increasing job well-being among men with children under the age of one. For women, supervisors must also take into consideration that they can complement and substitute the WLB alternatives offered by the firm to guarantee women's possibilities of balancing work and life.

The limitations of this study may give rise to further research. First, we studied a small number of practices to analyze WLB, and other ways to reconcile work and family life may have a higher impact on women's job-well-beings compared to flexible work arrangements. Future research could explore whether other human resource practices influence well-being and whether this influence is conditioned by SS. Second, although the data used in this research were compiled from 27 different countries, all countries were European. The Arabic world and Asian cultures, among other countries with idiosyncratic social values that are likely to differ from those of Western countries, were not considered in this study. Therefore, the results should not be generalized without first determining whether the cultural context helps explain women's job well-beings after childbirth. In addition, it could be interesting to replicate this study in other cultures.

## Author contributions

AL-C conceived and designed the research questions or hypotheses, performed the literature research on well-being and analyzed the data. AG-C performed the literature research on on female worker after a childbirth, analyzed the data and wrote discussion. LP-A performed the literature research on Work-life balance and women's perceived job well-being after childbirth. DC-M performed the literature research on on Supervisory support and women's perceived job well-being after childbirth.

### Conflict of interest statement

The authors declare that the research was conducted in the absence of any commercial or financial relationships that could be construed as a potential conflict of interest.

## Footnotes

^1^Associated Press in Madrid (2017, 10 April). Carme Chacón, Spain's first female defence minister, dies. https://www.theguardian.com/world/2017/apr/10/chacon-spain-first-female-defence-minister-dies.

^2^(2015, 20 April). United Nation for Entity for Gender Equality and the Empowerment of Women. http://www.unwomen.org/en/about-us/about-un-women.
